# Ultrasound-guided needle-knife for De Quervain's disease: A protocol for systematic review and meta-analysis

**DOI:** 10.1097/MD.0000000000024877

**Published:** 2021-04-09

**Authors:** Li Jiang, Huan Liu, Huaiyu Li, Jiawang Jiang, Xiaomin Liu

**Affiliations:** a Jiangxi University of Traditional Chinese Medicine; b Nanchang Hongdu Hospital of TCM, Nanchang, Jiangxi Province, PR China.

**Keywords:** De Quervain's disease, needle-knife, protocol, systematic review, ultrasound-guided

## Abstract

**Background:**

De Quervain's disease is a kind of aseptic inflammation caused by repeated frictions of tendons in the tendon sheath of the styloid process of the radius. The main symptoms are protuberance and pain of the styloid process of the radius, accompanied by aggravation of pain during the movement of the wrist and thumb. The advantages of needle-knife are simple operation, obvious therapeutic effect and high safety. It can also be used to treat De Quervain's disease. Ultrasound gives a precise visualization of the thickness. The purpose of this study is to evaluate the efficacy and safety of ultrasound-guided needle-knife in the treatment of De Quervain's disease and to provide the latest basis for clinical application.

**Methods:**

The computer will be used to search all randomized controlled trials (RCTs) about ultrasound-guided needle-knife treatment of De Quervain's disease in the following database: PubMed, Web of Science, Cochrane Library, Cochrane Central controlled Trials Registry (CENTER), EMBASE, China National knowledge Infrastructure (CNKI), Wanfang data, Chinese Biomedical Literature Database (CBM), VIP Database (VIP). The effectiveness and safety of ultrasound-guided needle-knife in the treatment of De Quervain's disease were evaluated with pain intensity, wrist function as the main index and wrist range of motion, adverse events and quality of life as the secondary index. Revman5.3 software was used for data processing.

**Results:**

This study will provide the latest evidence for the Ultrasound-guided needle-knife for De Quervain's disease.

**Conclusion:**

The conclusion of this study is to evaluate the effectiveness and safety of ultrasound-guided needle-knife in the treatment of De Quervain's disease.

**Unique INPLASY number:**

INPLASY202110094.

## 1 Introduction

De Quervain's disease, also known as radial styloid stenosing tenosynovitis, has a high clinical incidence, which is one of the common causes of pain and swelling on the radial side of the wrist.^[1]^ The recurrence rate of the disease is high, and due to the popularity of computers, mobile phones and other electronic products, the incidence of the disease tends to be younger. The repeated movement of the wrist joint causes the tendon of the first extensor compartment of the wrist joint to pass through the fibrous bone tunnel at the level of the styloid process of the radius, and the repeated sliding of the tendon leads to aseptic inflammation, fibrous degeneration and fibrous ossification and thickening of the tendon sheath, resulting in a series of aseptic inflammatory reactions in the tendon sheath.^[2,3]^

Local blocking therapy is often used to treat De Quervain's disease in clinic, and minimally invasive surgery is also used in severe cases, which has a certain curative effect but is often accompanied by adverse reactions, such as local subcutaneous tissue atrophy and local skin pigmentation.^[4]^ The traditional Chinese medicine treatments of De Quervain's disease are mainly non-operative treatment, including needle knife, acupuncture, external application of internal medicine, physiotherapy and so on, which have the characteristics of simple operation and high safety.

As one of the important methods of treating diseases in traditional Chinese medicine, the needle knife has been widely used in the treatment of tendon injuries. Compared with other treatments, the needle knife has the advantages of satisfactory curative effect, less side effects and low cost.^[5]^ At present, needle knife is mostly used to treat DeQuervain's disease, it can relieve pain and restore wrist function, which has been proved by some clinical trials.^[6]^ However, the needle knife treatment of De Quervain's disease commonly used manipulation techniques can lead to universal and unnecessary iatrogenic injuries. As an imaging modality, ultrasound has become widely accepted in the musculoskeletal system evaluation. It is quick, cheap, and easily available. Also, ultrasonography-guided therapy is real-time, non-invasive and non-radiative. By the guidance of ultrasound, problems such as deciding the depth or the possible injury of tissues will be solved.^[7,8]^ Although the effectiveness of ultrasound-guided needle-knife in the treatment of De Quervain's disease is optimistic, the evidence is limited, it is necessary to strictly review the evidence of ultrasound-guided needle-knife in the treatment of De Quervain's disease.^[9]^ Therefore, we decided to conduct this study to further evaluate the effectiveness and safety of ultrasound-guided needle-knife in the treatment of De Quervain's disease, and to provide clinical evidence.

## 2 Methods

### 2.1 Study registration

The review scheme of this study has been registered on the INPLASY website and registration number were INPLASY202110094 (URL https://inplasy.com/inplasy-2021-1-0094/). This study will be reported in accordance with the Preferred Reporting Project (PRISMA-P) Statement Guidelines for Systematic Reviews and Meta-Analysis Agreements.^[10]^

### 2.2 Type of studies

All randomized controlled trials (RCTs) on ultrasound-guided needle-knife for DeQuervain's disease will be included. Other designs, such as animal studies, case reports, reviews, and non-randomized controlled trials will be excluded. There are no restrictions on language and publication date.

### 2.3 Types of patients

All the patients included in the study were diagnosed as De Quervain's disease, regardless of age, sex, race and course of disease.

### 2.4 Types of interventions.

#### 2.4.1 Experimental interventions

The treatment group was treated with needle knife, which was not limited by acupoint selection, operation method, needle material, needle retention time, course of treatment and so on.

#### 2.4.2 Control interventions

There was no restriction of intervention in the control group. Studies of needle knife and other treatments were included in this study if other treatments were used in the treatment group as well as in the control group.

### 2.5 Types of outcome measures

#### 2.5.1 Primary outcomes

(1) pain intensity, such as visual analogue score (VAS); (2) wrist function, such as Cooney wrist score, Gratland and Werley wrist score, wrist function evaluation, and so on.

#### 2.5.2 Secondary outcomes

1. wrist range of motion;
2. adverse events;
3. quality of life.

#### 2.5.3 Exclusion criteria

1. RCTs comparing two different types of needle knife;
2. Non-randomised controlled trials;
3. Duplicated data;
4. Invalid outcome indexes.

### 2.6 Search strategy

#### 2.6.1 Electronic searches

We will search the following databases by computer: PubMed, Web of Science, Cochrane Library, Cochrane Central controlled Trials Registry (CENTER), EMBASE, China National knowledge Infrastructure (CNKI), Wanfang data, Chinese Biomedical Literature Database (CBM), VIP Database (VIP). Search from the establishment of the database to October 2020. Search for combinations of subject words and free words. Search terms include ultrasound, needle-knife, De Quervain's disease, radial styloid stenosing tenosynovitis, trigger-finger and random allocation. There are no restrictions on language, country and population. The complete search strategy of PubMed is shown in Table 1. And similar search strategy will be applied to other electronic databases.

**Table 1:** . Search strategy used in PubMed database.

Order	Search items
#1	(“De Quervain's disease"[Mesh]) OR ((((( wrist and hand's tenosynovitis) OR ( Trigger finger)) OR ( Stenosing tenosynovitis)) OR ( De Quervain's tenosynovitis)) OR (Trigger-finger)[All Fields]
#2	((((((((((((((((Acupuncture) OR (acupuncture therapy)) OR (Electroacupuncture)) OR (electroacupuncture therapy)) OR (manual acupuncture)) OR (moxibustion)) OR (Acupuncture, Ear)) OR (Acupunctures, Ear)) OR (Ear Acupunctures)) OR (Auricular Acupuncture)) OR (Ear Acupuncture)) OR (Acupuncture, Auricular)) OR (Acupunctures, Auricular)) OR (Auricular Acupunctures)) OR (warm acupuncture)) OR (fire needling)) OR (elongated needle) [All Fields]
#3	(“ultrasound” [Mesh]) OR ((((ultrasound wave) OR (sonography)) OR (supersonic wave)) OR (ultrasonic wave)) [All Fields]
#4	randomized controlled trial [Publication Type] OR randomized [Title/Abstract] OR placebo [Title/Abstract]
#5	#1 AND #2 AND #3 AND #4

#### 2.6.2 Searching other resources

We also search articles related to needle-knife and De Quervain's disease to replace or supplement some reference lists, such as systematic reviews. At the same time, search conference papers and related clinical trial registries, such as the World Health Organization (WHO) International Clinical trial Registry ((ICTRP)), the US National Institutes of Health Clinical trial Registry, Australia and New Zealand Clinical trial Registry and China Clinical trial Registry.

## 3 Analysis

### 3.1 Data collection

#### 3.1.1 Studies selection

**Figure 1:**
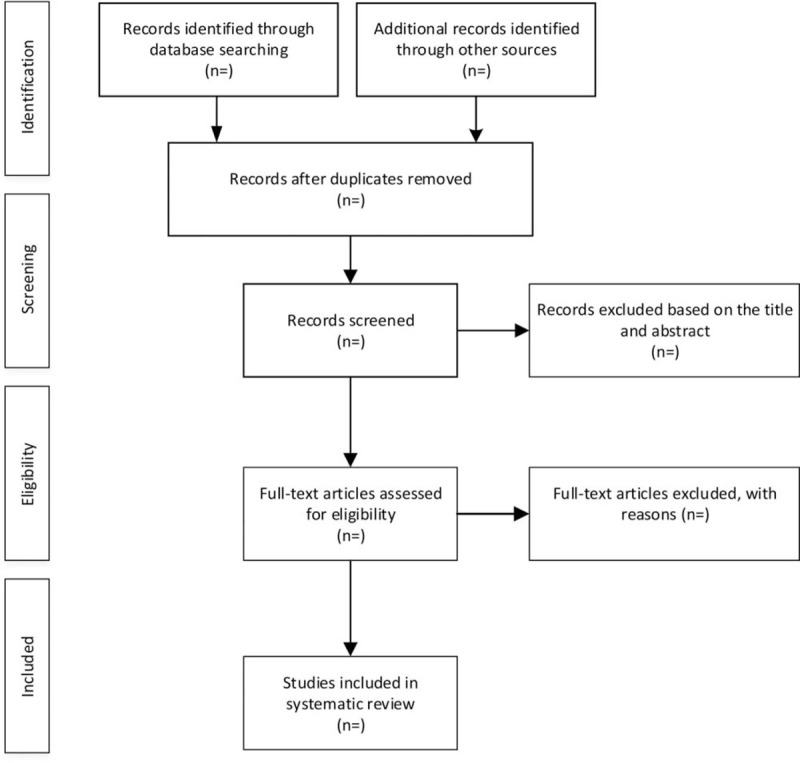
Flow chart of study selection.

The researchers imported all documents from electronic searches and other sources into EndnoteX7 software to deduplicate data. We delete obvious unqualified articles by reading titles and abstracts. Then read the full text of the remaining articles, and contact the author for details, group discussion to determine the final inclusion of the literature. Literature retrieval and screening are carried out independently by two researchers at the same time, and when there is a difference of opinion, it is settled by another research member through consultation, and the final literature included is examined. Details of the selection procedure for studies are shown in a PRISMA flow diagram (Fig. 1).

#### 3.1.2 Data extraction and management

The two researchers used pre-designed tables to collect data, collect the following information: study data (first author or newsletter author, publication time, study type), subject details (baseline data, diagnostic criteria, exclusion criteria, sex, age), research methods (sample size, randomization, blind method, distribution concealment, etc.), intervention measures in the treatment group and the control group. Details of needle knife treatment (needle knife method, acupoint selection, course of treatment), result measurement (primary and secondary results, follow-up, adverse events). Any differences in the data collection process will be resolved through discussion by a third researcher. If the above data in the article is incomplete, we will contact the author to supplement it.

### 3.2 Assessment of risk of bias in included studies

In the aspect of inclusion of literature bias risk, Cochrane Collaboration's bias risk tool will be used to evaluate the quality of inclusion.^[11]^ It includes the following seven items: random sequence generation; allocation concealment; blinding of participants; blinding of outcome; incomplete outcome data; selective result report; and other sources of bias. All of the above projects will be evaluated independently by two researchers. If there are differences, we will resolve them by discussing or consulting a third researcher. The bias risk of each project is rated as low, high or unclear risk.

### 3.3 Measures of treatment effect

RevMan software was used for comprehensive analysis. The results of dichotomy data are expressed by the risk ratio of 95%CI, and the continuous data are expressed by mean difference (MD) or standardized mean difference (SMD).

### 3.4 Missing data dealing with

If the research data is lost, the author will be contacted by email or telephone to obtain the appropriate information. If the data we need is not available, it will be analyzed through the existing data to assess whether it is included in the study.

### 3.5 Assessment of heterogeneity

*X*^2^ test according to Cochrane manual, *P* <.10 will be considered significant. At the same time, the *I*^2^ value will be calculated. If *I*^2^ ≤ 50%, the statistical heterogeneity in this study is acceptable, and the effect will be estimated by the fixed-effects model. If *I*^2^ > 50%, there is significant heterogeneity, using random-effects model.

### 3.6 Publication bias

When more than 10 trials were included in this study, the funnel chart was used to judge the report bias. If there is asymmetry in the funnel diagram, the Egge test of Stata software will be used for quantitative analysis.

### 3.7 Data synthesis

We will use Revman5.3 software for statistical analysis. First of all, to judge whether there is statistical heterogeneity between the results, if there is statistical heterogeneity, the source of heterogeneity should be analyzed. After excluding the influence of obvious clinical heterogeneity, random-effects model should be used for meta-analysis. If not, the fixed-effects model is used for analysis. If there is significant clinical heterogeneity, subgroup analysis or sensitivity analysis are performed.

### 3.8 Subgroup analysis

Classifications are as follows:

1. different needle knife methods
2. different acupoints
3. different courses of treatment.

### 3.9 Sensitivity analysis

We will eliminate the “high-risk” low-quality articles for sensitivity analysis to judge the robustness of the results.

### 3.10 Ethics and dissemination

Ethical approval is not required for this study because individual patient data are not needed in our study. The results of our study will be published in peer-reviewed journals.

## 4 Discussion

De Quervain's disease is a common chronic motor system injury disease, which often causes pain in the wrist joint and aggravates during activity. Whose basic pathology is chronic aseptic inflammation caused by repeated friction of abductor pollicis and extensor pollicis brevis in the tendon sheath, which affects people's daily life, which is more common in housewives, manual operators and workers who use mobile phones and computers for a long-time.^[12,13]^ The treatment of this disease includes conservative treatment and surgical treatment. And local blocking therapy is a common way to treat the disease, intrathecal injection of steroids in local pain to achieve the purpose of local anti-inflammatory, but hormone injection may lead to local skin pigmentation, local subcutaneous tissue atrophy, elevated blood sugar complications.^[14,15]^

The needle knife is widely used in treating De Quervain's disease. But there are still some drawbacks. The doctor can observe and analyze the characteristics of the tissue and muscle, and improve the effect and safety of the operation by using ultrasound-guided.^[16–18]^ The combined use of ultrasound and the needle knife get a better treatment for De Quervain's disease.^[19,20]^ There is no systematic scientific evaluation for ultrasound-guided needle-knife at the present time, so the article aims to provide evidence-based medical evidence on the safety and effectiveness of ultrasound-guided needle-knife.

## Author contributions

**Conceptualization:** Li Jiang, Xiaomin Liu.

**Data curation:** Huan Liu.

**Formal analysis:** Li Jiang, Huaiyu Li.

**Methodology:** Li Jiang, Huan Liu.

**Software:** Xiaomin Liu.

**Supervision:** Jiawang Jiang.

**Writing – original draft:** Huaiyu Li, Jiawang Jiang.

**Writing – review & editing:** Huaiyu Li, Jiawang Jiang.
